# Topical Tacrolimus as a Novel Treatment for Refractory Idiopathic Facial Aseptic Granuloma in a Pediatric Patient

**DOI:** 10.7759/cureus.104657

**Published:** 2026-03-04

**Authors:** Terry Henry, Grace Staes, Shivam Gulhar, Mohannad Al-Samarraie, Nikisha Richards

**Affiliations:** 1 Department of Ophthalmology, Virginia Commonwealth University, Richmond, USA; 2 Department of Ophthalmology, Howard University College of Medicine, Washington, D.C., USA

**Keywords:** calcineurin inhibitor, eyelid inflammation, granulomatous rosacea, idiopathic facial aseptic granuloma (ifag), pediatric eyelid lesion, refractory chalazion, tacrolimus

## Abstract

This report describes the case of a four-year-old girl with idiopathic facial aseptic granuloma (IFAG) who developed persistent, treatment-resistant eyelid and facial inflammation despite multiple courses of antibiotics and corticosteroids. Her presentation included recurrent hordeola, severe blepharitis, and a cheek nodule, with a clinical picture more consistent with IFAG than pilomatrixoma despite histologic evidence of the latter. Standard therapies provided only transient benefit and were limited by systemic side effects. Initiation of topical tacrolimus 0.03% ointment led to rapid and sustained improvement in redness, irritation, and nodularity. This case highlights topical tacrolimus as a promising non-steroidal option for children with prolonged or refractory IFAG.

## Introduction

Idiopathic facial aseptic granuloma (IFAG) is a rare granulomatous condition affecting primarily children aged three to four years, with a slight female predominance [1]. IFAG is classically underrecognized and likely underreported. A retrospective observational study done at two general hospitals in Buenos Aires, Argentina, reported only 43 cases between 2004 and 2022 [2]. Clinically, IFAG manifests as solitary, asymptomatic nodules localized to the cheek, outer eyelid, or perioral regions [3]. 

The term “aseptic” denotes the noninfectious nature of the granulomatous inflammation, and therefore incision and drainage are not usually recommended, as they are not abscesses. IFAG primarily affects young children and adolescents, with a mean age of presentation of approximately four years, and exhibits a female predominance, with an estimated female-to-male ratio of 2:1. Clinically, IFAG may be mistaken for other periocular and facial lesions, including chalazion, hordeolum (stye), and pyogenic granuloma [3]. A chalazion represents a chronic, sterile, lipogranulomatous inflammation secondary to obstruction of the Meibomian gland and typically presents as a firm, painless, slow-growing nodule. In contrast, a hordeolum is an acute, painful, erythematous lesion resulting from a staphylococcal infection of the sebaceous or Meibomian glands, often with a visible pustular point. Pyogenic granuloma, a rapidly proliferating vascular lesion composed of granulation tissue, generally arises following local irritation, trauma, or a prior chalazion and presents as a reddish, pedunculated mass on the conjunctiva [4]. 

The precise pathogenesis of IFAG remains unclear, though several theories have been postulated. Some authors hypothesize a traumatic origin, whereas others propose an inflammatory reaction associated with embryological remnants of the skin adnexa. Others postulate that IFAG is a pediatric manifestation of granulomatous rosacea due to its frequent association with chalazia and eyelid inflammation [5]. 

In an analysis of case reports involving IFAG, Neri et al. explore whether IFAG should be considered part of the granulomatous rosacea spectrum in children. All patients showed clinical improvement with systemic macrolide antibiotics (clarithromycin or erythromycin) and/or topical metronidazole, therapies commonly used in rosacea. Microbiologic cultures were negative, reinforcing the aseptic, inflammatory nature of the lesions. The association with chalazia, similar histopathologic features, and response to rosacea-directed therapies support the hypothesis that IFAG represents a pediatric variant of granulomatous rosacea, rather than a distinct infectious or abscess-like entity [6]. 

Histologically, IFAG is characterized by granulomatous infiltration without evidence of infectious organisms [3]. Diagnosis is typically made clinically, as imaging and biopsy are seldom required [1]. Most cases follow a benign and self-limited course with spontaneous resolution within nine to 12 months [1,7]. However, due to the cosmetic and symptomatic impact of lesions, treatment is often initiated. First-line interventions commonly include conservative therapies such as warm compresses, topical metronidazole, or systemic antibiotics like macrolides [5,7]. While metronidazole is frequently employed for its localized anti-inflammatory properties, its efficacy is often inconsistent in more deep-seated or recalcitrant granulomatous presentations. This clinical limitation necessitates the consideration of more targeted immunomodulators, such as tacrolimus. As a topical calcineurin inhibitor, tacrolimus disrupts T-lymphocyte activation to halt the production of pro-inflammatory cytokines [8]. It serves as a valuable corticosteroid-sparing agent, offering a potent anti-inflammatory response without the risk of skin thinning or atrophy associated with traditional steroids [9]. Given emerging data on the efficacy of topical calcineurin inhibitors in granulomatous and rosacea-like dermatoses, we present a case of a young female patient with an atypically prolonged and treatment-resistant case of IFAG, ultimately responsive to topical tacrolimus, suggesting its potential as a novel therapeutic approach in refractory presentations.

## Case presentation

**Figure 1:**
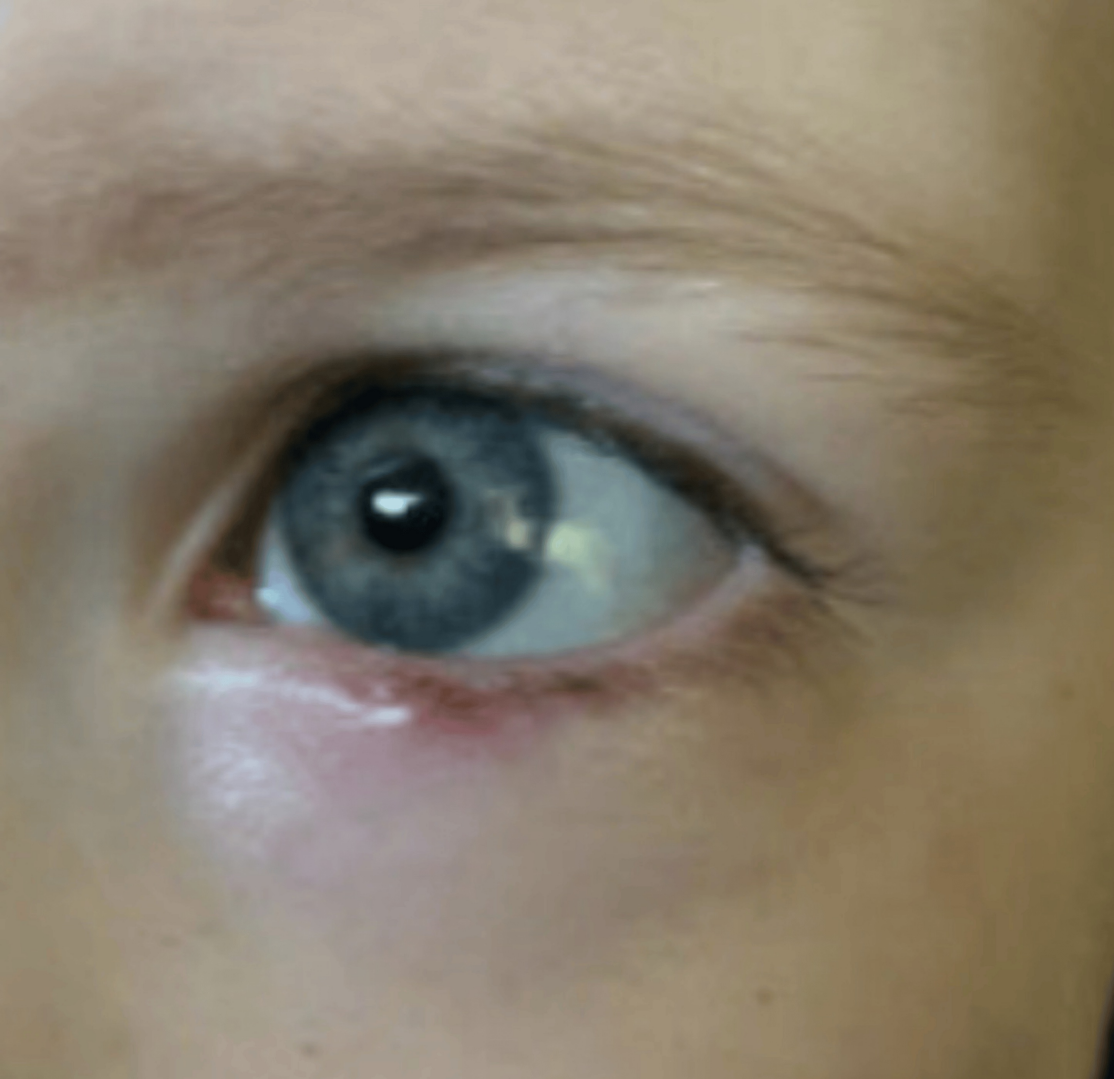
An external photograph of the right lower eyelid demonstrates a lower eyelid hordeolum. The lesion appears as a localized, erythematous swelling near the lid margin.

A four-year-old female patient without any significant medical history was referred for evaluation of a persistent eyelid lesion. Initially, she had developed a right lower eyelid hordeolum and a longer-standing upper lid chalazion on the same side, which were treated conservatively with lid scrubs, warm compresses, and omega-3 supplementation (Figure 1). 

**Figure 2:**
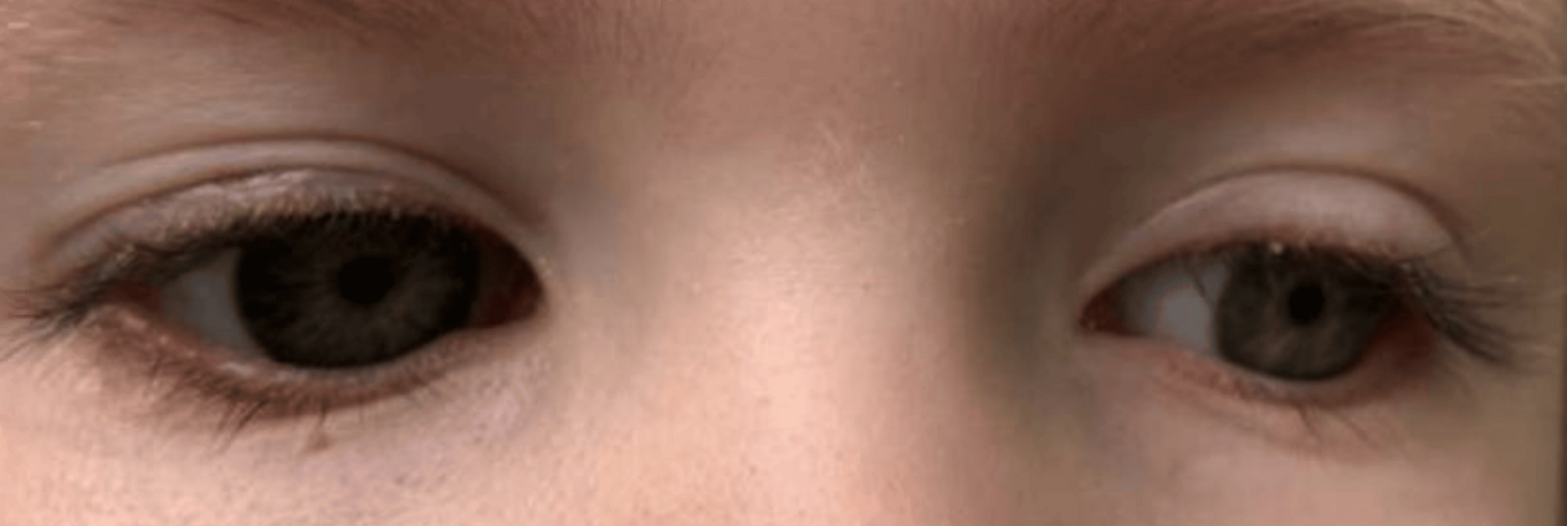
External photograph of both eyes showing severe blepharitis (grade 4+) involving the upper and lower eyelids bilaterally. Marked lash debris and eyelid margin inflammation are evident. A previously present external hordeolum of the right lower eyelid had resolved at the time of examination, though signs of active lid margin disease persist.

Despite initial management, the condition progressed to include recurrent hordeola and increasingly severe blepharitis involving both upper and lower eyelids (Figure 2). Her symptoms included persistent swelling, nightly discharge, matted lashes, and eventually, photophobia and irritation of both eyes.

**Figure 3:**
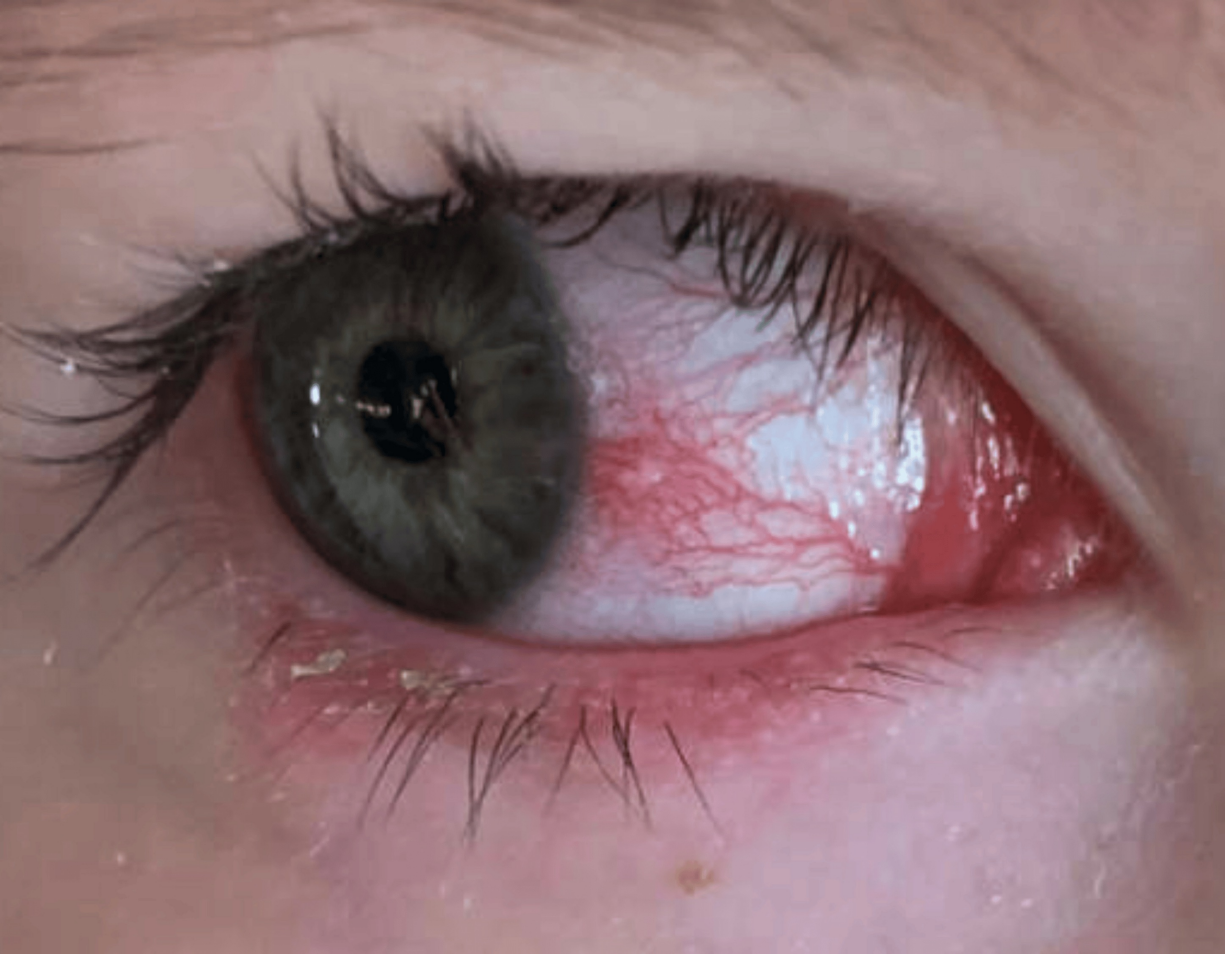
The external photograph of the right eye demonstrates crusting of the lashes, conjunctival injection, and an upper eyelid hordeolum. The patient was clinically diagnosed with blepharokeratoconjunctivitis, with slit-lamp examination revealing peripheral corneal infiltrates and ongoing ocular surface inflammation secondary to eyelid margin disease.

Examination over time revealed confluent external hordeola of both the upper and lower lids, dense lash collarettes, conjunctival injection, Horner-Trantas dots, and signs of bilateral blepharokeratoconjunctivitis (Figure 3). Treatment regimens were escalated sequentially, including the addition of prednisolone acetate 1% drops, neomycin/polymyxin B/dexamethasone ophthalmic ointment, oral clarithromycin, and both oral and compounded topical metronidazole. While some treatments offered temporary improvement, the patient continued to experience flares, eyelid erythema, and worsening inflammation.

On repeat examination during a concurrent dermatologic evaluation, a solitary, well-demarcated, dome-shaped nodule was noted on the left infraorbital region. The lesion measured approximately 1 cm in diameter and exhibited a distinct erythematous to violaceous hue with a smooth overlying surface. This presentation occurred at the 1.5-year mark of a chronic, fluctuating disease course. Initial management for concurrent vernal keratoconjunctivitis (VKC) involved 10 months of conservative therapy, including warm compresses, prednisolone acetate 1% ophthalmic suspension, and omega-3 supplementation. While the VKC resolved, the facial lesion persisted.

The appearance of this distinct granuloma prompted a 14-day course of neomycin, polymyxin B, and dexamethasone (Maxitrol) twice a day (BID); a planned 14-day course of oral clarithromycin; and a two- to four-month trial of topical metronidazole BID. However, the clinical course was complicated by treatment intolerance, as oral clarithromycin was discontinued after only two days. At a two-month follow-up, the facial granuloma remained refractory. Subsequent trials of oral metronidazole were initiated to manage a new hordeolum, blepharoconjunctivitis, and corneal infiltrates, but these were also aborted within 24 hours due to systemic intolerance. While the acute ocular findings resolved three months later, the facial granuloma persisted.

Notably, four months after the ocular resolution, a new atypical lesion appeared on the contralateral right cheek. A biopsy of this secondary lesion suggested a pilomatrixoma, but this histopathology did not correspond to the morphology or clinical behavior of the primary left-sided infraorbital lesion. Given the prolonged 22-month disease course and refractoriness to multiple therapies, the patient was referred to oculofacial plastic surgery. Following assessment and discussion regarding the rare association between IFAG, childhood rosacea, and eczema, treatment with topical tacrolimus 0.03% ointment was initiated on the affected area.

Within one week of initiating tacrolimus therapy, there was a marked reduction in redness, irritation, and blepharitis. Although the patient experienced transient blurred vision due to the ointment base, the medication was well-tolerated overall. Over the following months, flare-ups became infrequent. The patient’s mother reported significant improvement in ocular comfort, along with the complete resolution of eyelid erythema and nodularity.

## Discussion

IFAG, though rare, is an important consideration in the differential diagnosis of pediatric patients presenting with persistent or atypical facial or eyelid lesions [1,3]. While its clinical presentation is generally benign, IFAG’s resemblance to more common or infectious eyelid conditions such as chalazia, hordeola, or pilomatrixoma often leads to delays in diagnosis or misdirected treatment [3]. In our case, a biopsy of the facial lesion revealed a diagnosis of pilomatrixoma. However, the discrepancy between these two diagnoses underscores the fundamental differences in their clinical presentation, anatomic distribution, imaging characteristics, and histopathologic features, even though both may present as facial nodules in pediatric patients.

Pilomatrixoma is a benign tumor derived from hair follicle matrix cells and typically presents as a hard, calcified, slowly enlarging subcutaneous mass, most commonly affecting the head, neck, or upper extremities in children and adolescents [10]. On ultrasound, pilomatrixomas characteristically demonstrate calcifications, and histologic examination reveals basophilic cells, shadow (anucleate keratinized) cells, foreign body giant cells, and frequently areas of calcification or ossification [11]. While pilomatrixoma was confirmed histologically, it typically presents as a solitary, firm, non-recurrent nodule that does not respond to anti-inflammatory therapy. Furthermore, recurrence, waxing and waning inflammation, and multifocal eyelid involvement are uncommon in pilomatrixoma, making IFAG the more likely clinical diagnosis [12].

In contrast, IFAG presents as a painless, non-calcified inflammatory nodule, classically localized to the cheek in younger children, with a mean age of presentation of approximately 3.8 years [13]. Ultrasound typically demonstrates a well-demarcated hypoechoic lesion without calcium deposits, directly contrasting with the calcified appearance seen in pilomatrixoma [14]. Histologically, IFAG is characterized by a chronic dermal lymphohistiocytic granulomatous infiltrate with foreign body-type giant cells but lacks the defining shadow cells and calcification seen in pilomatrixoma [8]. Clinically, IFAG follows a waxing and waning, self-limited course with spontaneous resolution over several months, rather than the persistent growth pattern observed in pilomatrixoma [8,13].

In our patient, the broader clinical picture, marked by recurrent blepharitis, episodic inflammatory eyelid nodules, and chronic facial inflammation, was not consistent with the typical behavior of pilomatrixoma, which usually presents as a solitary, firm, non-recurrent nodule that does not respond to anti-inflammatory therapy [12]. In contrast, IFAG is characterized by a waxing and waning course, inflammatory features, and occasional eyelid involvement, making it a more appropriate unifying diagnosis for this patient’s constellation of symptoms. In this context, pilomatrixoma may represent a coincidental or secondary histopathologic finding rather than the primary driver of disease. Additionally, the proposed association between IFAG and granulomatous rosacea further complicates diagnostic interpretation and may reflect shared inflammatory pathways influencing both clinical appearance and therapeutic response [7].

Management of IFAG is not standardized due to its relative infrequency and benign nature. Many cases resolve spontaneously within several months, and mild presentations may warrant observation alone [1]. In symptomatic or cosmetically concerning cases, treatment options have historically included topical metronidazole, systemic macrolides, or antiparasitic agents [5,7]. While these therapies are usually effective, there exists a subset of patients, such as the one described here, for whom the disease proves prolonged and resistant to standard treatment. These cases underscore the need for alternative therapeutic options.

Tacrolimus, a calcineurin inhibitor, applies its immunosuppressive effects by blocking T-cell activation and inhibiting the transcription of pro-inflammatory cytokines such as interleukin-2. It has shown benefit in conditions with dysregulated cutaneous immune responses, including atopic dermatitis and steroid-refractory rosacea [15]. Its off-label use in IFAG is not well documented, making this case notable for demonstrating its efficacy in a patient who failed to respond to both corticosteroids and antibiotics.

The immunologic etiology of IFAG is unclear, but the favorable response to tacrolimus supports the hypothesis that immune dysregulation plays a key role in disease pathogenesis. Given the chronic inflammatory nature of the lesion and the histologic evidence of granulomatous infiltration, the success of tacrolimus suggests that downregulating local immune activity may be a valuable treatment strategy in refractory cases [16]. Importantly, topical tacrolimus offers a non-steroidal alternative, potentially reducing the risk of steroid-induced ocular side effects such as elevated intraocular pressure, cataracts, and dermal atrophy [6].

This case contributes to a growing body of anecdotal evidence advocating for a broadened treatment paradigm in IFAG. While large-scale studies are currently lacking, this report highlights the potential for tacrolimus to serve as an effective and well-tolerated therapy in children who experience extended or complicated courses of IFAG.

## Conclusions

This case highlights the potential role of topical tacrolimus as an alternative treatment in pediatric patients with prolonged, treatment-resistant IFAG. In the face of incomplete response to antibiotics and corticosteroids, tacrolimus offered significant symptom relief and disease control. Future studies exploring its efficacy in a broader cohort may further inform its clinical utility in IFAG management.
